# Transurethral resection of the prostate versus prostatic artery embolization in the treatment of benign prostatic hyperplasia: a meta-analysis

**DOI:** 10.1186/s12894-019-0440-1

**Published:** 2019-01-28

**Authors:** Yu-Li Jiang, Lu-Jie Qian

**Affiliations:** 1 Department of Urology, The Affiliated Hospital of Hang Zhou Normal University, Hangzhou, 310015 China; 2 School of Medicine, Hang Zhou Normal University, Hangzhou, 310016 China

**Keywords:** Benign prostatic hyperplasia, Transurethral resection of the prostate, Prostatic artery embolization, Meta-analysis

## Abstract

**Background:**

To compare the clinical efficiency and safety of transurethral resection of the prostate (TURP) and prostatic artery embolization (PAE) for the treatment of Benign prostatic hyperplasia (BPH).

**Methods:**

We searched PUBMED, EMBASE and the Cochrane Central Register for studies from May 1998 to May 2018 for studies comparing the efficiency and safety of TURP with PAE. Four studies met the inclusion criteria for our meta-analysis. After data extraction and quality assessment, we used RevMan 5.2 to pool the data.

**Results:**

A total of four studies involving 506 patients were included in our meta-analysis. The pooled data showed that the Qmax was higher in TURP group than PAE with a significant difference (WMD:4.66, 95%CI 2.54 to 6.79, *P* < 0.05). The postoperative QOL was lower in the TURP than PAE group (WMD: -0.53, 95%CI -0.88 to − 0.18, *P* < 0.05). The postoperative prostate volume was significantly smaller in the TURP than PAE group (WMD: -8.26, 95%CI -12.64 to − 3.88, *P* < 0.05). The operative time was significantly shorter in the TURP than PAE group (WMD: -10.55, 95%CI -16.92 to − 4.18, *P* < 0.05). No significant difference was found in the postoperative IPSS and complications between TURP and PAE (*P* > 0.05, WMD:1.56, 95%CI -0.67 to 3.78, *p* = 0,05, OR:1.54, 95%CI 1.00 to2.38, respectively).

**Conclusions:**

TURP could achieve improved Qmax and QoL compared to PAE. Therefore, for patients with BPH and lower urinary tract symptoms (LUTS), TURP was superior to PAE.

## Background

Benign prostatic hyperplasia (BPH) is common among elderly males, affecting 50% at the age of 60 years [1, 2]. The main manifestations of BPH are intermittently decreased urinary stream, nocturia and urgency, which are also called lower urinary tract symptoms (LUTS), and BPH accounts for 60% of LUTS in 50–60-year-old men [1]. Treatment options for BPH often include medical waiting, minimally invasive approaches, alpha-1-Blocker, or surgical therapies. Medical treatment, which has been considered first-line treatment, can include the use of 5-reductase inhibitors; these inhibitors have been demonstrated to reduce prostate volumes by more than 30 ml [3]. Surgical treatments (transurethral resection of the prostate or open surgery) are used for treatment in the event of failed medical management [4]. Transurethral resection of the prostate (TURP) is considered the gold standard of treatment for men with prostate volumes of 30–80 ml, and it is also appropriate for those with prostate volumes > 80 ml [1, 5]. However, the complications of TURP include sexual dysfunction, retrograde ejaculation, postoperative hemorrhage, continent urinary retention, TURP syndrome and urinary stricture [6, 7]. Prostatic artery embolization (PAE) has been suggested as a minimally invasive interventional radiological procedure [8]. In PAE, injection of microspheres or small particles or directly into the prostatic arteries bilaterally or unilaterally could result in ischemia or an enlarged prostate [9]. Michlle et al. first reported that embolization of hypogastric arteries (PAE) could be used to control severe prostate hemorrhage caused by BPH [10].

Several studies had compared the clinical outcomes and safety of TURP with PAE [11–14]. Schreuder et al. reported a systematic review of the use of TURP and PAE for treating BPH [15]. Ray et al. recently reported an observational study comparing the efficiency and safety of TURP and PAE for treating BPH [13]. Feng et al. reported a meta-analysis of 20 studies about PAE for treating BPH [16]. However, no meta-analysis was previously performed to compare the clinical efficiency and safety of TURP and PAE.

At present, no standards or guidelines exist for evaluating the clinical efficacy of TURP and PAE for treating BPH. Therefore, we thought it necessary to perform a meta-analysis to evaluate the optimal treatment by comparing the clinical outcomes and safety between TURP and PAE. The purpose of this study is to compare the efficiency and perioperative safety with the quantitative data for the clinical efficacy and safety of PAE or TURP in treating BPH.

## Methods

### Search strategy

This meta-analysis was performed according to the Preferred Reporting Items for systemic Reviews and meta-analysis (PRISMA) statement. We searched PUBMED, EMBASE and the Cochrane Central Register for relevant studies published in English from May 1998 to May 2018. We used the following search terms: “BPH”, “benign prostate hyperplasia”, “prostatic arterial embolization”. We also used the combined Boolean operators “AND” or “OR” in the Title/Abstract.

### Inclusion and exclusion criteria

**Fig. 1:**
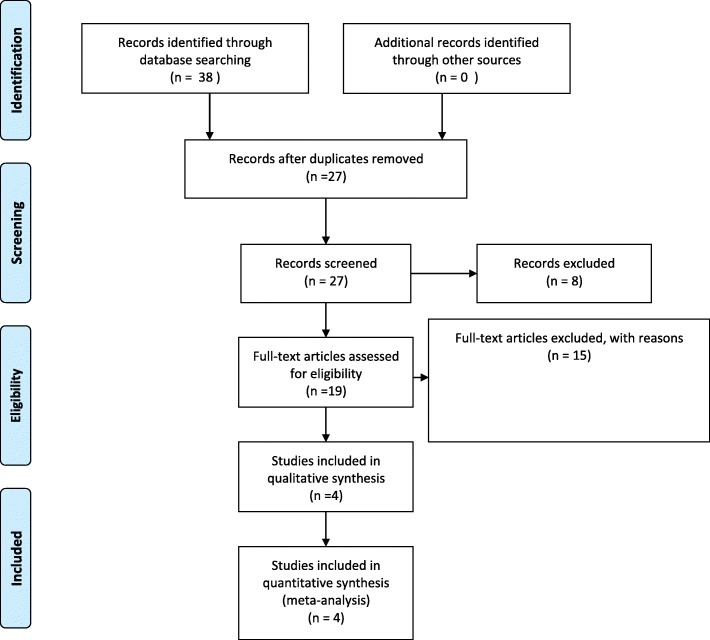
Flow diagram of the process for the selection of relevant studies

The two investigators reviewed the articles. The inclusion criteria were as follows: (1) comparative analysis of TURP and PAE for treating BPH; (2) BPH patients treated with TURP and PAE; (3) follow-up duration longer than 12 months; and (4) articles with the IPSS, Qmax, Qol, postoperative prostate, volume, and operative time. The exclusion criteria were as follows: (1) case reports, reviews, editorial comments, meeting abstracts and articles without applicable data; (2) studies with insufficient data, such as missing the SD (standard deviation) and number and (3) studies that were not comparative in nature. The process of identifying relevant studies is summarized in Fig. 1.

### Data extraction

Two authors reviewed the relevant studies. The two authors extracted data, such as the IPSS, Qmax, and QOL score. The following data were recorded: (1) baseline comparative data; (2) clinical outcomes; and (3) postoperative complications. Two reviewers (YLJ and LJQ) assessed the quality of the included studies.

### Statistical analysis

We used Review Manager Version 5.2 software with the Mantel-Haenszel method (The Cochrane Collaboration, Oxford, UK) and Stata 12.0 to perform the analysis of the included data. We used Cochran’s Q to evaluate the heterogeneity; Q < 50% or *P* > 0.01 was indicative of little heterogeneity. However, Q > 50% or *P* < 0.01 was indicative of heterogeneity. For quantitative data, we used the weight mean difference (WMD) and 95% confidence interval (CI) or standard mean difference (SMD) and 95%CI to calculate continuous data. We used the odds ratio (OR) and 95%CI to evaluate binary data. The statistical significance level was 0.05.

## Results

Four studies were included in our study [11–14]. The process of obtaining these studies is summarized in Fig. 1. From the selected databases, 38 studies were obtained. After screening the titles and abstracts, 11 studies were excluded. After detailed processing, 23 studies were excluded. Finally, 4 studies were included in our meta-analysis. Table 1 summarizes basic characteristics of the included studies.

**Table 1:** Basic characteristics of the included studies

Study	Year	Design	IPSS		prostate volume(ml)		Study group	
			TURP	PAE	TURP	PAE	TURP(n)	PAE(n)
Gao	2014	RCT	23.1	22.8	63.5	64.7	57	57
Qiu	2017	P, S	23.9	24.5	68.7	64.6	40	17
Ray	2018	R, M	21.63	21.3	101.2	65.6	216	89
Carnevale	2016	RCT	27.6	25.3	56.6	63.0	15	15

*P* Prospectively study, *RCT* Randomised controlled trial, *S* Sigle center, *R* Retrospectively study, *M* Mutli-centers

### Quality assessment of the included studies

We used the New-Ottawa Scale (NOS) to evaluate the included studies. The NOS scores were evaluated using a 9-point system. An NOS score of 7 or above is considered as higher quality, and an NOS score of 3 or below was believed to have a lower quality. For RCT, we assessed the risk of bias according to the Cochrane Collabortation handbook, version 5.0. Table 2 shows quality assessment of the included studies.

**Table 2:** Quality assessment of the included studies

Study Design		Selection				Comparability	Outcome			Total
		Representativeness of exposed cohort	Selective of nonexposed Cohort	Ascertainment of exposure	Outcome not present at start		Assessment of outcome	Adequate follow-up length	Adequacy of follow-up	
Qiu	P, S	^*^	^*^	^*^	^*^	^*^	^*^	^*^	^*^	8
Ray	R, M	^*^	^*^	^*^	^*^	^*^	^*^	^*^	^*^	8
		Randomization	Allocation concealment		Blinding			Quality level		
Carnevale	RCT	Adequate	Unclear		Unclear			Unclear		
Gao	RCT	Adequate	Unclear		Unclear			Unclear		

*P* Prospectively study, *RCT* Randomised controlled trial, *R* Respectively study, *M* mutli-centers

The symbol "*" represents score

### IPSS

**Fig. 2:**
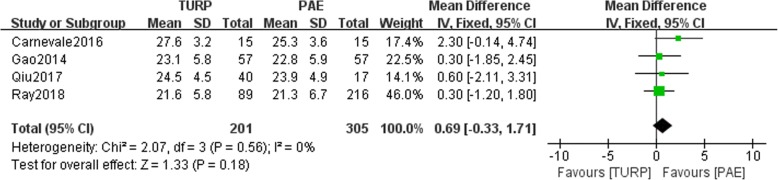
Forest plot for the preoperative IPSS between the TURP and PAE for BPH

**Fig. 3:**
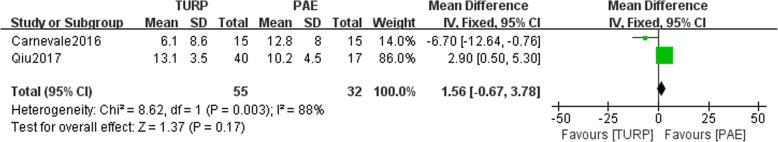
Forest plot for the postoperative IPSS between the TURP and PAE for BPH

The pooled data of preoperative IPSS indicated no statistical differences between the TURP group and PAE group (*n* = 506, WMD: 0.69, 95%CI: -0.33 to 1.71, I^2^ = 0; *p* = 0.18, fixed-effects model, Fig. 2). Data related to the postoperative IPSS were available in two studies No significant difference between the TURP and PAE groups was noted (*n* = 87, WMD: 1.56, 95%CI: -0.67 to 3.78, I^2^ = 88%; *p* = 0.17, fixed-effects model, Fig. 3).

### Qmax

**Fig. 4:**
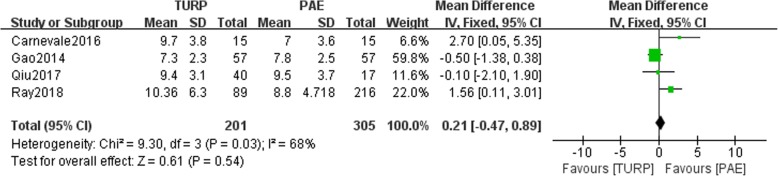
Forest plot for the preoperative Qmax between the TURP and PAE for BPH

**Fig. 5:**
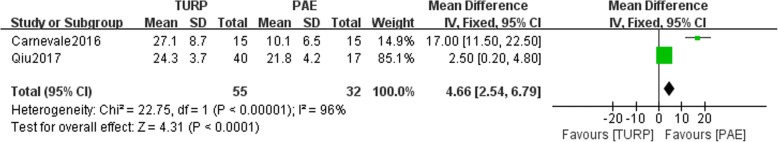
Forest plot for for the postoperative Qmax between the TURP and PAE for BPH

The pooled data of preoperative Qmax indicated no statistical differences between the TURP group and PAE group (*n* = 506, WMD: 0.21, 95%CI: -0.47 to 0.89, I^2^ = 68%; *p* = 0.54, fixed-effects model, Fig. 4). The postoperative Qmax was higher in the TURP group than in the PAE group (*n* = 87, 55 patients were in the TURP group, 32 patients were in the PAE group, WMD: 4.66, 95%CI: 2.54 to 6.79, I^2^ = 96%, *P* < 0.05, fixed-effects model, Fig. 5).

### Qol

**Fig. 6:**
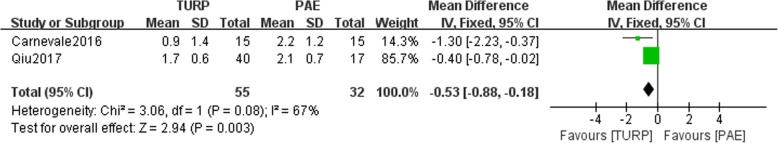
Forest plot for Qol between the TURP and PAE for BPH

Two studies reported the Qol. The Qol was lower in the TURP group than in the PAE group (n = 87; 55 patients were in the TURP group and 32 patients were in the PAE group, WMD; − 0.53, 95%CI; − 0.88 to − 0.18, I^2^ = 67%; *p* < 0.05, fixed-effects model, Fig. 6).

### Prostate volume

**Fig. 7:**
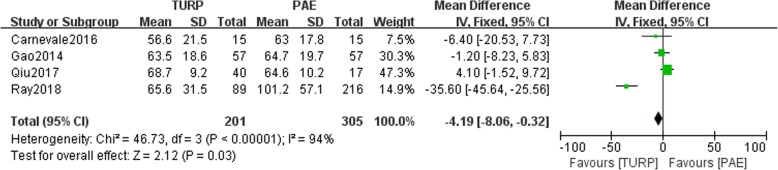
Forest plot for the preoperative prostate volume between the TURP and PAE for BPH

**Fig. 8:**
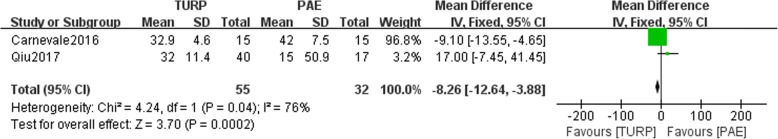
Forest plot for for the postoperative prostate volume between the TURP and PAE for BPH

The pooled data of preoperative prostate volume indicated statistical differences between the TURP group and PAE group (*n* = 506, WMD: -4.19, 95%CI: -8.06 to − 0.32, I^2^ = 94%; *p* = 0.03, fixed-effects model, Fig. 7). Data related to the postoperative prostate volume were available in two studies. The postoperative prostate volume was smaller in the TURP group than in the PAE group (*n* = 87; 55 patients were in the TURP group and 32 patients were in the PAE group, WMD; − 8.26, 95%CI; − 12.64 to − 3.88, I^2^ = 76%; *p* < 0.05, fixed-effects model, Fig. 8).

### Operative time

**Fig. 9:**
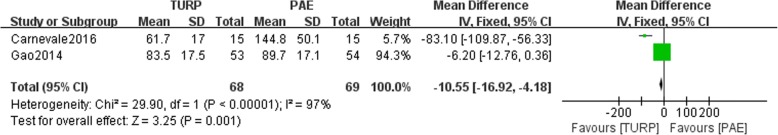
Forest plot for operative time between theTURP and PAE for BPH

Two studies reported the operative time. The operative time was shorter in the TURP group than in the PAE group (*n* = 137; 68 patients were in the TURP group and 69 patients were in the PAE group, WMD; − 10.55, 95%CI; − 16.92 to − 4.18, I^2^ = 97%; *p* = 0.001, fixed-effects model, Fig. 9).

### Complications

**Fig. 10:**
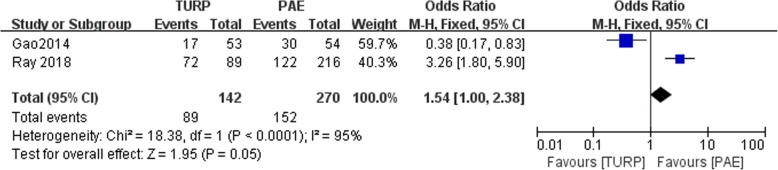
Forest plot for complications between the TURP and PAE for BPH

Two studies reported complications. No statistically significant difference between the TURP and PAE groups was noted (*n* = 412, 142 patients were in the TURP group, 270 patients were in PAE group, OR; 1.54, 95%CI; 1.00 to 2.38, I^2^ = 95%; *p* = 0.05, fixed-effects model, Fig. 10).

## Discussion

In the present study, we included four clinical studies with at least a 12-month follow-up. The present systematic review and meta-analysis was performed to compare the efficiency of TURP and PAE for the treatment of BPH. In addition, our study was the first meta-analysis to compare the efficiency and safety of TURP and PAE for the treatment of BPH.

Our meta-analysis demonstrated that patients with BPH had comparable basic characteristics. The present study compared the short-term follow-up outcomes among patients undergoing TURP versus PAE. These patients shared comparable common baseline characteristics. In our study, the IPSS and complications were not significantly different for patients undergoing TURP versus PAE. The Qol, Qmax, operative time, and postoperative volume were better in TURP than in PAE.

Feng et al. performed a meta-analysis that reported only one method for the efficiency and safety of PAE [16]. Their meta-analysis indicated that the IPSS and Qol score showed great improvement after PAE (*P* < 0.05). However, this study did not compare the TURP and PAE groups. Schreuder et al. published a review about the management of BPH [15].

In present study, no statistically significant difference in the preoperative Qmax between the 2 groups (*p* > 0.05, Fig. 4). The Qmax could be as a parameter to compare the clinical efficacy between the TURP group and PAE group. We found that the postoperative Qmax was higher in the TURP group than in the PAE group (P < 0.05, Fig. 5). This may be from the TURP achieving a radical resection of an enlarged volume. It may take longer after PAE to achieve the histopathologic changes associated with destruction of the prostate vasculature [17]. However, the PAE could not make a big change for non-radical removal of the prostate volume. Similarly, Carnevale et al. reported that the Qmax of patients in the TURP group was higher than in the PAE group. In our study, the Qmax of patients had a better change after PAE, which was related to apoptosis in the prostate tissue. Bagla et al. reported a single-arm trial involving 18 patients who received PAE, demonstrating that the Qmax was decreased in the sixth-month follow up compared with the first and third months [18]. This may also be attributed to the recurrence of new enlarged prostate tissue.

The pooled studies showed statistically significant difference in the preoperative prostate volume between the 2 groups (*p* > 0.05, Fig. 7). This may due to the selction bias among the four studies. Our study demonstrated that the residual prostate volume was smaller in the TURP group than in the PAE group. The direct resection of pathologically protruding prostate tissue could instantaneously achieve satisfactory urodynamics outcomes. The PAE group could have a later decrease in the prostate volume. Other studies reported a similar outcome [14, 19]. Pisco et al. reported that the postoperative prostate volume was associated with clinical efficiency [20]. Amouyal et al. reported that improvement of urodynamics was not associated with the clinical outcome [21]. Tinto et al. reported a single-arm PAE trial wherein the PVR significantly improved after PAE. They reported on 103 patients undergoing PAE for BPH [22]. They demonstrated that the PVR was much changed after PAE.

The pooled studies showed no statistically significant difference in the preoperative IPSS between TURP group and PAE group (*p* > 0.05, Fig. 2). Our study reported that the two groups could achieve a similar IPSS score for BPH treatment (*p* > 0.05, Fig. 3). Qiu et al. reported that the TURP group had a lower IPSS score than the PAE group (*P* = 0.021) [12]. Similarly, Carnevale et al. reported that the score was lower for patients in the PAE group than in the TURP group. Additionally, the posttreatment IPSS was lower for patients in the TURP group than for patients in the PAE group (*p* = 0.012 and *p* = 0.0007, respectively) [14]. Pisco et al. reported improvement of 20 ml/s in the Qmax and 12 points in the IPSS score together [20].

With respect to the Qol, the TURP group had a lower score than the PAE group, demonstrating that the TURP group could achieve a higher life quality. Ray et al. reported that the Qol score changed from baseline of 14.4 to 12 for the last follow-up, which was 2.00 in PAE, and the score changed from 4.9 in the beginning to 1.5 in the last follow-up [13]. Tinto et al. reported that the Qol score improved slightly from 4.11 to 2.01 at 12 months. This observation changed little in the next 12 months.

Our study showed no statistically significant difference in complications between the TURP and PAE groups (Fig. 10). However, Gao et al. demonstrated that the complication rate was higher after PAE [11]. In addition to blood transfusion, acute urinary retention was higher in the PAE group. Grosso et al. reported that studies on PAE described few complications, while bleeding, sexual dysfunction, and incontinence were more common with TURP [23].

The operation time was shorter in the TURP group than in the PAE group. This may be from unfamiliarity with the complicated anatomy. Wang et al. reported that the artery of prostate was a variant [24]. Additionally, TURP, unlike PAE, was a routine surgical method to treat with BPH for most centers.

Our study also had several limitations. First, not all included studies were RCTs, which may decrease the quality of evidence. Additionally, the sample size of patients was low. Second, publication and selection bias should also be considered when interpreting the results. Third, the included studies had a shorter follow-up period. This may contribute to worse outcomes for the PAE group. PAE may require a long follow-up time. Additionally, the studies that included PAE may have different embolization standards. This design may contribute to bias. Moreover, the four studies involved unilateral embolization, bilateral artery embolization or combined unilateral and bilateral embolization, which also increases the heterogeneity. We could not eliminate all sources of heterogeneity.

## Conclusions

In our meta-analysis, the efficiency of TURP for treating BPH was better than that of PAE. Our meta-analysis shows that PAE is an efficient and safe procedure that achieves better improvement in urodynamics and Qol. Our meta-analysis indicates that TURP is superior to PAE in clinical efficiency improvement. More multi-center high quality RCTs with large sample size are needed to verify the clinical efficiency of TURP and PAE for the treatment of BPH.

## Abbreviations

BPH: Benign prostatic hyperplasiaI
CI: Confidence interval
IIEF: International index of erectile function
IPSS: International prostate symptom score
LUTS: Lower urinary tract symptoms
OR: Odds ratio
PAE: Prostatic arterial embolization
PVR: Postvoid residual volume
Qmax: Maximum urinary flow rate/peak urinary flow rate
QoL: Quality of life
TURP: Transurethral resection of the prostate
WMD: Weight mean difference
